# Macrophage Polarization: Different Gene Signatures in M1(LPS+) vs. Classically and M2(LPS–) vs. Alternatively Activated Macrophages

**DOI:** 10.3389/fimmu.2019.01084

**Published:** 2019-05-24

**Authors:** Marco Orecchioni, Yanal Ghosheh, Akula Bala Pramod, Klaus Ley

**Affiliations:** ^1^Division of Inflammation Biology, La Jolla Institute for Immunology, La Jolla, CA, United States; ^2^Department of Bioengineering, University of California, San Diego, La Jolla, CA, United States

**Keywords:** macrophage, innate immunity, M1, M2, cancer

## Abstract

Macrophages are found in tissues, body cavities, and mucosal surfaces. Most tissue macrophages are seeded in the early embryo before definitive hematopoiesis is established. Others are derived from blood monocytes. The macrophage lineage diversification and plasticity are key aspects of their functionality. Macrophages can also be generated from monocytes *in vitro* and undergo classical (LPS+IFN-γ) or alternative (IL-4) activation. *In vivo*, macrophages with different polarization and different activation markers coexist in tissues. Certain mouse strains preferentially promote T-helper-1 (Th1) responses and others Th2 responses. Their macrophages preferentially induce iNOS or arginase and have been called M1 and M2, respectively. In many publications, M1 and classically activated and M2 and alternatively activated are used interchangeably. We tested whether this is justified by comparing the gene lists positively [M1(=LPS+)] or negatively [M2(=LPS–)] correlated with the ratio of *IL-12* and *arginase 1* in transcriptomes of LPS-treated peritoneal macrophages with *in vitro* classically (LPS, IFN-γ) vs. alternatively activated (IL-4) bone marrow derived macrophages, both from published datasets. Although there is some overlap between *in vivo* M1(=LPS+) and *in vitro* classically activated (LPS+IFN-γ) and *in vivo* M2(=LPS–) and *in vitro* alternatively activated macrophages, many more genes are regulated in opposite or unrelated ways. Thus, M1(=LPS+) macrophages are not equivalent to classically activated, and M2(=LPS–) macrophages are not equivalent to alternatively activated macrophages. This fundamental discrepancy explains why most surface markers identified on *in vitro* generated macrophages do not translate to the *in vivo* situation. Valid *in vivo* M1/M2 surface markers remain to be discovered.

## Introduction

Macrophages are critical cells in both tissue homeostasis and inflammation, performing essential tissue-specific functions as well as protecting the organism from infection. Their origin has been debated extensively during the past years (1, 2). It is now clear that most tissue macrophages arrive before definitive hematopoiesis is established. Monocytes derive from a common progenitor called Macrophage Dendritic Cell Precursor (MDP), emphasizing a continuum differentiation potential of monocytes to both inflammatory macrophages and DCs (3). However, although monocytes undoubtedly can become macrophages, data from recent studies have challenged the generalized applicability of this dogma. Sathe et al. also challenged the idea that MDPs are the restricted progenitors of monocytes and macrophages. Thus, they show that MDPs can differentiate into other hematopoietic lineages (4). Other studies nicely summarized by Florent Ginhoux and Martin Guilliams (1) reported that most tissue macrophages in mice are not generated from monocytes in the steady state. Instead, mature tissue macrophages are derived from embryonic precursors that seed the tissues before birth, maintaining their numbers in adults by self-renewal (1). Primitive macrophages first appear within the yolk sac blood islands, then spread into the tissues of the embryo through the blood as soon as the circulatory system is established, giving rise to the fetal macrophage populations. These cells maintain a gene expression signature different from bone marrow-derived macrophages (BMDM). However, whether yolk sac-derived macrophages can persist into adulthood giving rise to specific adult tissue macrophage populations is still debated (5). Of note, all lineage tracing studies of tissue macrophages were completed in mice. While it is reasonable to assume that human tissue macrophages also derive from embryonic precursor cells, this hypothesis is difficult to test rigorously.

Since the discovery of tissue-specific gene signatures in peritoneal macrophages (6), many published transcriptomic data show that each tissue macrophage set has both tissue-specific and shared gene signatures, based on the tissue of origin (7). The tissue-specific gene signatures will not be discussed further. They need to be taken into consideration when comparing macrophages in different parts of the body.

Tissue macrophage transcriptomes dramatically change after transfer to a tissue culture environment (8). Gosselin et al. show that mouse and human microglia transferred to a tissue culture environment gain and lose expression of hundreds of genes. Preferential reduction in expression of microglia-specific genes such as *MAF, RUNX*, and *SMAD3* was documented (8, 9). Thus, there appears to be a specific “*in vitro*” macrophage transcriptome, reflecting the fact that the cells were removed from their normal tissue environment and placed in a tissue culture dish. Most published macrophage work uses this *in vitro* setting, most commonly for bone marrow-derived macrophages (BMDM) for mouse macrophages and monocyte-derived macrophages (MDM) for human macrophages. The *in vitro* environment and the different stimuli used to differentiate the cells such as CSF1 (M-CSF) and CSF2 (GM-CSF) have substantial polarizing effects. These colony-stimulating factors are known to “prime” or “activate” macrophages as well as induce their differentiation (10). Studies comparing M-CSF and GM-CSF in BMDM show that they can activate different pathways: GM-CSF leads to a more pro-inflammatory state (TNF expression), and M-CSF induces a tissue healing state (IL-10 expression) after LPS stimulation (10). *In vitro*, macrophages change their polarization state based on diverse stimuli such as cytokines, microbes, microbial products, and other modulators (11).

In the literature, there are several terms and definitions to describe the macrophage activation and polarization. Mills defined M1 and M2 in Balb/c and C57BL/6 mice (12). Nathan defined classical macrophage activation by IFN-γ (13), Gordon defined alternative macrophage activation by IL-4 (14), Anderson and Mosser defined regulatory macrophages (Mreg) (15), Kadl defined a macrophage phenotype induced by oxidized lipids (Mox) (16), Gleissner defined the transcriptome of macrophages differentiated in the presence of CXCL4 (PF4, M4) (17), and Mantovani further subdivided alternatively activated macrophages into M2a,b,c (18).

The M1/M2 macrophage polarization nomenclature was introduced in 2000, based on the propensity of C57BL/6J macrophages to be more easily activated to produce NO (M1 polarized) than Balb/c mice (M2 polarized) (12). Different metabolism of arginine after LPS injection elicits different phenotypes of macrophages in C57BL/6J and Balb/c mice. C57BL/6J peritoneal macrophages induced iNOS resulting in nitric oxide and a T-helper 1 (Th1) CD4 T cell response, while Balb/c mice induced arginase to produce ornithine and a Th2 response. In analogy to Th1 and Th2, these macrophages were named M1 and M2. Subsequent work showed that M1 macrophages have a pro-inflammatory phenotype with pathogen-killing abilities and M2 macrophages promote cell proliferation and tissue repair (19). These findings suggested that macrophages from different strains of mice had a different propensity to produce NO and arginase in response to LPS.

We rigorously tested macrophage polarization using the hybrid mouse diversity panel (HMDP) generated by the Lusis group (20, 21). The HMDP is a panel of 83 inbred mouse strains, employed as a surrogate model for human immune diversity. Most bioinformatics tools are not designed to dissect a spectral distribution in a heterogeneous population. Analysis of commonly regulated genes after LPS treatment among all 83 strains yielded an empty set (no genes). Therefore, we established a gene expression-based factor that represents the degree of LPS-induced polarization (polarization factor). Since IL-12 is the known Th1 polarizing cytokine and arginase is the hallmark enzyme of M2 macrophages, we used the ratio of IL-12b/arginase-1 gene expression as a continuous parameter to rank all 83 mouse strains along the M1/M2 axis. Peritoneal macrophage transcriptomes were used to rank the 83 mouse strains based on their response to LPS (22). We found a continuous spectrum of LPS-induced activation: M1-polarized mice responded by upregulating many pro-inflammatory genes that were positively correlated with IL12/arginase. In accordance with Murray's recommendation to list the stimulus in parentheses (11), the M1 signature is synonymous with (=LPS+), i.e., genes whose expression show a positive correlation with the IL-12/arginase ratio in response to LPS. M2-polarized mice responded by upregulating genes that were negatively correlated with IL12/arginase (=LPS–) (22). A similar spectrum of responses was seen in two human macrophage datasets (22), suggesting that the HMDP is a valid way to reflect human macrophage diversity.

Cytokines can polarize macrophages *in vitro*. The classical activation is induced by LPS and IFN-γ. Classically activated macrophages are often called M1, but this is misleading, as our analysis will show. LPS activates oxidative metabolism and antimicrobial activity of macrophages (23). In the 1990s Gordon's group reported that the Th2 cytokine IL-4 increased the expression of the macrophage mannose receptor (MR, also known as CD206), which is normally down-regulated by IFN-γ, inducing an alternative activation of macrophages that promoted an anti-inflammatory and pro-healing phenotype (14, 24). Other cytokines such as IL-10 and TGF-β were also linked with macrophage polarization *in vitro*. Mantovani et al. called classically activated macrophages (by IFN-γ combined with LPS or tumor necrosis factor [TNF]) M1. *In vitro* alternatively activated macrophages (by IL-4) were re-named M2a. Two other M2-like macrophage phenotypes were induced by Fc receptor engagement by immune-complexes (M2b) or by IL-10 and glucocorticoids (M2c) (18). Based on Mantovani's work, many researchers started to use M1 for classical activated and M2 for alternatively activated macrophages *in vitro*. Unfortunately, that led to more confusion than clarification in the field.

With the final purpose to better understand the differences and similarities between *in vivo* and *in vitro* macrophage response, in this review, we compared mouse macrophage transcriptomes *in vivo* extracted from Buscher et al. (22) (GSE38705) to *in vitro* transcriptomes of bone marrow derived macrophages BMDMs that were either classically (LPS+IFN-γ, GSE69607) or alternatively (IL-4, GSE69607) activated (25).

## Analysis Criteria of *in vivo* and *in vitro* Signatures and Overview

The *in vitro* polarization signatures used in this review as described in Table 1 were obtained from Jablonski et al. (GSE69607) transcriptomic data and defined by comparing the differently expressed (DE) genes of *in vitro* alternatively activated BMDMs (IL-4, 20 ng/ml for 24 h) vs. the *in vitro* classically activated BMDMs (LPS, 100 ng/mL + IFN-γ 20 ng/mL for 24 h). All genes with log2 fold change (FC) >1 were considered in the *in vitro* alternatively activated macrophage (Mφ) signature; all genes with log2 FC < -1 were considered in the *in vitro* classically activated Mφ signature. An FDR < 0.05 were applied in this analysis (Supplementary Table 1).

**Table 1:** Macrophage transcriptomes overview.

**Original Manuscript**	**Jablonski et al. (25)**	**Buscher et al. (22)**
Macrophages	Bone marrow-derived, BMDM	Peritoneal macrophages
Activation	LPS (100 ng/mL) +IFN-γ (20 ng/mL), 24 h for Classically activated;	LPS (2 ng/mL) for 4 h
	IL-4 (20 ng/mL), 24 h for Alternatively activated.	
Transcriptome	Affymetrix, GSE69607	Affymetrix, GSE38705 Orozco et al. (21)
LPS source	Sigma-Aldrich L2880	List Biological Inc., Campbell, CA
Gene lists	DE between classically activated vs. alternatively activated, filtered for unique genes.	Positively [M1(=LPS+)] or negatively [M2(=LPS–)] correlated with IL-12/arginase-1

To further increase the significance of our analysis we filtered the two *in vitro* full signatures with the gene sets derived from *in vitro* classically activated (LPS+IFN-γ) and alternatively activated (IL-4) transcriptomes vs. untreated BMDMs transcriptome, respectively (GSE69607). All the DE genes passing the FDR < 0.05 cutoff were considered and used for the further analysis and discussions (Supplementary Table 2).

The signatures of *in vivo* bacterial LPS positive responders [M1(=LPS+)] vs. negative responders [M2(=LPS–)] peritoneal macrophages as established in Buscher et al. (22) (GSE38705) were used (Supplementary Table 3). Since the *in vitro* classically activated (LPS+IFN-γ treated) and alternatively (IL-4-treated) activated Mφ transcriptomes were derived from C57BL/6J mice, we filtered the full *in vivo* signatures specifically for the C57Bl/6J strain from LPS treated and untreated mice by listing the differently up- and downregulated genes (FDR < 0.05) between control and LPS-treated samples for the C57BL/6J strain only (20, 21) (Supplementary Table 4). The two newly originated *in vivo* macrophage signatures M1(=LPS+)C57BL/6J, M2(=LPS–)C57BL/6J, and the *in vitro* classically activated (LPS+IFN-γ treated) and alternatively activated (IL-4-treated) signatures were then analyzed and compared using the Venny 2.1 online tool (http://bioinfogp.cnb.csic.es/tools/venny/index.html).

**Figure 1:**
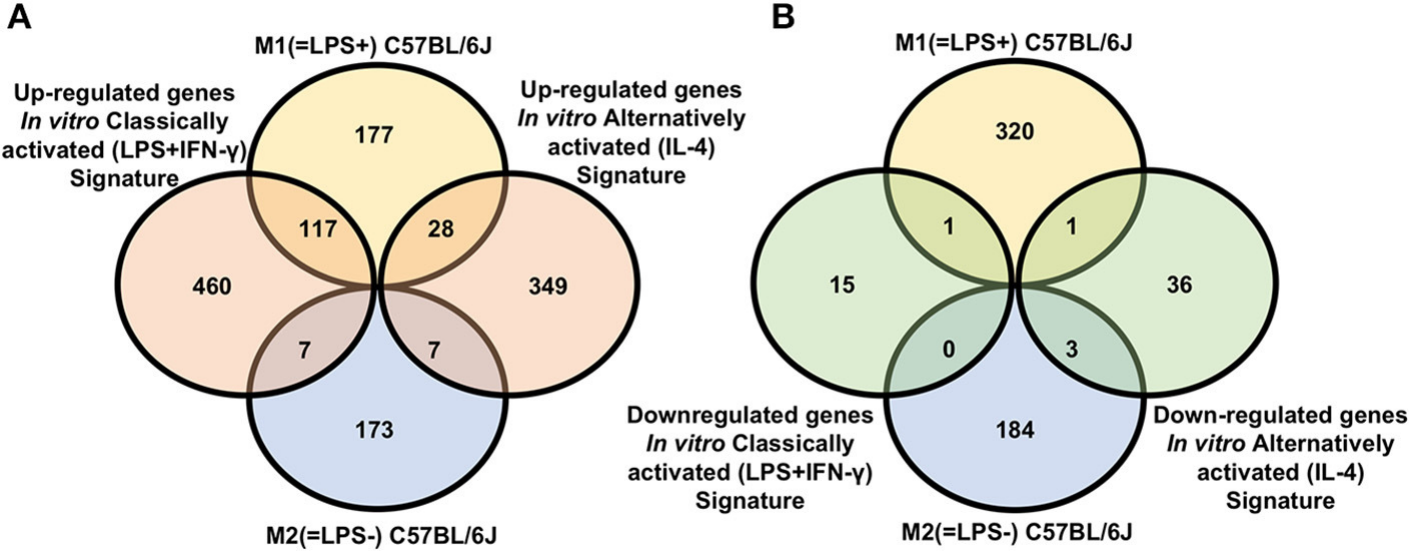
Signature comparison for C57BL/6 macrophages. **(A)** Venn diagram showing overlap between genes whose expression was positively [M1(=LPS+)C57BL/6J] or negatively [M2(=LPS–)C57BL/6J] correlated with IL12/arginase ratio *in vivo* with genes upregulated *in vitro* classically activated (LPS+IFN-γ) macrophages (left) or *in vitro* alternatively activated (IL-4) (right) macrophages vs. unstimulated. **(B)** Venn diagram showing overlap between genes whose expression was positively [M1(=LPS+)C57BL/6J] or negatively [M2(=LPS–)C57BL/6J] correlated with IL12/arginase *in vivo* with genes downregulated *in vitro* classically activated (LPS+IFN-γ) macrophages (left) or *in vitro* alternatively activated (IL-4) (right) macrophages vs. unstimulated.

Of the 322 M1 (=LPS+) genes positively correlated with IL-12/arginase, 117 were also upregulated in *in vitro* classically activated (LPS+IFN-γ) Mφ, but 28 were also upregulated in *in vitro* alternatively activated (IL-4) Mφ. Conversely, of the 186 genes in the *in vivo* M2(=LPS–)C57BL/6J signature whose expression was negatively correlated with IL12/arginase, seven each were shared with *in vitro* classically and alternatively activated (IL-4) Mφ (Figure 1A, Supplementary Table 5). Strikingly, 460 genes were private to *in vitro* classically activated (LPS+IFN-γ) Mφ and 349 genes were private to *in vitro* alternatively activated (IL-4) Mφ, respectively. One thousand seven hundred seventy-six genes were private to M1(=LPS+)C57BL/6J and 173 were private to *in vivo* M2(=LPS–)C57BL/6J. Since *in vivo* M1 is widely considered synonymous with *in vitro* classically activated (LPS+IFN-γ) and M2 is considered synonymous with *in vitro* alternatively activated (IL-4) Mφ, these discrepancies are striking. Only very few genes (15) were downregulated *in vitro* classically activated (LPS+IFN-γ) Mφ compared to unstimulated Mφ, or in alternatively activated (IL-4) Mφ compared to unstimulated Mφ (36 genes, Figure 1B, Supplementary Table 5). There was almost no overlap (total of five genes) between the gene set genes) between the gene set downregulated by activation *in vitro* and the *in vivo* gene set M1/M2(=LPS±) positively or negatively correlated with IL-12/arginase (Figure 1B). Since M1(=LPS+)C57BL/6J is considered the opposite of *in vitro* alternatively activated (IL-4) and M2(=LPS–)C57BL/6J is considered the opposite of classically activated, we will focus on the differentially expressed private genes that are not shared, because they point out the difference between *in vivo* and *in vitro*.

### *In vivo* and *in vitro* M1 Signatures

#### *In vivo* M1(=LPS+)C57BL/6J and *in vitro* Classically Activated (LPS+IFN-γ) Mφ Share a Pro-inflammatory Response Backbone

Among the 117 genes upregulated in both *in vivo* M1(=LPS+)C57BL/6J and *in vitro* classically activated (LPS+IFN-γ) Mφ (Figure 1A, Supplementary Table 5) are many interferon-induced genes. Among interferon-regulated genes were *Irf9, Irf7, Ifi35, Ifnar2, Isg20, Ifit2*, and *Ifih1*. Interferon regulatory factors actively regulate macrophage activation and polarization with the expression of pro-M1 genes such as IL-12 (*Il12a* and *Il12b*) (26, 27). The expression of these genes is mediated by the activation of Janus kinase (*Jak2*) JAK/signal, transducer and activator of transcription signaling pathway guided by signal transducer and activator of transcription (*Stat1/Stat2*) (28).

Costimulatory molecules such as *Cd86* and *Cd40* relevant to antigen presentation were also represented among both *in vivo* M1(=LPS+)C57BL/6J and *in vitro* classically activated (LPS+IFN-γ) Mφ signatures (29–31). CD86 is a ligand for CD28 and is a known inflammatory Mφ/DC marker (32). CD40 is a member of the TNF receptor superfamily (*Tnfrsf5*) and involved in antibody isotype switching and vascular inflammation (33). Another interesting gene expressed is *Cd38*, described by Jablonski et al. (25) to be a good marker for *in vitro* classical macrophage discrimination. Here we found that *Cd38* upregulation is shared among signatures. However, the exact functional role of CD38 in macrophages is still unclear. If *Cd38* surface expression is correlated with *Cd38* gene expression, CD38 might be a good new marker for M1 polarization (34).

Chemokines and their receptors are the main players in recruiting leukocytes during an active immune response. Cytokine receptors shape their function. Shared between the M1 and classically activated signatures we found *Cxcl16, Cxcl9, Il15ra*, and *Il17ra*. The chemokines *Cxcl16* and *Cxcl9* are known to support M1 polarization mostly primed by IFN stimulation (35). A previous study has shown that IL-15R alpha expression on macrophages supports the early transition of antigen-specific effector CD8(+) T cells to memory cells (36).

The TNF receptor-associated factor (*Traf1*) expression, together with TNF receptor superfamily members like *Tnfrsf1b* suggest that both M1 and classically activated macrophages respond strongly to TNF-like signals. Pro-inflammatory cytokine expression is well-known to increase adhesion molecule expression such as ICAM-1 in endothelial cells and macrophages in an NFκB-dependent manner (37). When integrin molecules on the leukocyte surface bind endothelial ICAM-1, this leads to outside-in signaling. *In vivo* M1(=LPS+)C57BL/6J and *in vitro* classically activated (LPS+IFN-γ) Mφ share upregulation of VCAM-1 (*Vcam1*) and ICAM-1 (*Icam1*). Recently, it was suggested that *Icam1* may regulate macrophage polarization by inhibiting the M2 polarization in tumor-associated macrophages (TAM) (38).

Intriguingly, Poly(ADP-Ribose) Polymerase Family Member 9 (Parp9) is also in the list of genes shared between *in vivo* M1(=LPS+)C57BL/6J and *in vitro* classically activated (LPS+IFN-γ) Mφ. Parp9 is a critical activator of macrophages (39). Modulating its expression has been shown to inhibit the activation of macrophage and can ameliorate atherosclerosis in mouse models (Patent US20160289685).

#### *In vivo* M1(=LPS+)C57BL/6J Exclusive Genes Drive Anti-bacterial Immune Response

The *in vivo* M1(=LPS+)C57BL/6J signature contains 177 uniquely up-regulated genes (Figure 1A, Supplementary Table 5). Compared to the *in vitro* signatures we found the expression of many genes correlated with the LPS mediated activation of Toll-like receptor 4 (*Tlr4*). Downstream of *Tlr4* is myeloid differentiation factor 88 (*MyD88*), *Nfkb1, Rela, Rps6ka2, Tank*, and *Ripk2*, (40) all expressed in M1(=LPS+)C57BL/6J. These genes are responsible for an anti-bacterial immune response. The chemokine *Ccl2* and the hematopoietic cytokine *Csf2* (GM-CSF) are positively correlated with M1 polarization *in vivo*, but not increased in response to IFN-γ and LPS *in vitro*. The same is true for the pro-inflammatory cytokines *Il15, Il23a, Irf1*, and *Ifnb1*.

Several pro-inflammatory cytokines, such as TNF and IL-12 have been reported to induce *Csf2*, whereas IL-4 and IL-10 suppress *Csf2* (41). Its expression enhances macrophage differentiation toward a pro-inflammatory state by activating the expression of cytokines (i.e., IL-23) and chemokines such as *Ccl2*, promoting leukocyte recruitment (42). IL-23a is part of the IL-12 family that includes other structurally related, heterodimeric cytokines such as IL-12, IL-27, and IL-35 (43). IL-23a drives inflammation through the induction of IL-17, promoting a highly pro-inflammatory Th17 response (44).

IL-15 is up-regulated in macrophages in response to LPS (45, 46). IL-15 was described to induce Th1 and Natural Killer cell (NK) immune responses by inducing IFN-γ and the transcription factor T-bet (47). Interferon regulatory factors in macrophages like *Irf1* actively regulate a macrophage effector activation with the expression of other pro-M1 genes such as IL-12 (*Il12a* and *Il12b*) and iNos (*Nos2*) (27).

Modulation of genes involved in general cellular metabolic activities is a prominent feature of macrophage differentiation and polarization. M1 macrophages mostly activate catabolic processes whereas M2 is skewed toward anabolic metabolism. The *in vivo* M1(=LPS+)C57BL/6J signature indeed includes genes responsible for an active protein catabolic process such as *Psme2*. Psme2 and other members of the Psme family are related to proteasome modulation. Proteasome modulation regulates macrophage function (48). The metabolism of proteins and amino acids (such as arginine) is fundamental for the correct activation of macrophages. The LPS stimulation indeed can induce the metabolism of arginine to NO and citrulline (49). This effect is mediated by different genes such as the cationic amino acid transporters *Slc7a2, Slc12a4, Slc1a4, Slc39a14, Slc3a2*, and *Slc4a7* found expressed exclusively in the *in vivo* M1(=LPS+)C57BL/6J signature.

#### *In vitro* Classically Activated (LPS+IFN-γ) Mφ Exclusively Activate Chemotaxis and Cell Migration Functions

The *in vitro* classically activated (LPS+IFN-γ) Mφ signature present after activating BMDMs with LPS and IFN-γ includes many genes involved in chemotaxis and cell migration (Figure 1A, Supplementary Table 5). Chemokines found in the *in vitro* classically activated (LPS+IFN-γ) Mφ signature include *Cxcl1* and *Cxcl2*, which recruit neutrophils (50). *Ccl5* (Rantes), a potent chemoattractant for T Cells, is also present. Other chemokines and chemokine receptors such as *Ccl3, Cxcl10, Cxcl11, Ccl25, Cx3cr1*, and *Ccr7* are in the *in vitro* signature. It also includes many cytokines well-known in classically activated macrophages: IL-1α, IL1β, IL-6, and TNF (*Il1a, Il1b, Il6*, and *Tnf*).

TNFR-associated factors (TRAFs) and other TNF induced proteins (*Tnfaip*) are important mediators of innate immune receptor signaling. *Traf2* and *Tnfaip3* are expressed in the classically activated macrophages *in vitro*, their function mostly related to regulating TLR signaling and mediating the response of other TNFR family members. *Traf2* and *Tnfaip3* induce proinflammatory cytokines and type I interferons in response to LPS or other stimuli (51).

Fifteen genes are down-regulated in classically activated macrophages but increase with LPS in M1 macrophages (Figure 1B, Supplementary Table 5). Many of them relate to the endocytosis process such as *Adrb2, Aif1, Apoc2, Coro1a, Sorl1*, and *Sirpb1a* (51).

### *In vivo* and *in vitro* M2 Signatures

#### *In vivo* M2(=LPS–)C57BL/6J and *in vitro* Alternatively Activated (IL-4) Mφ Share Proliferation, Apoptosis, and Differentiation Induced Genes

Among the *in vivo* M2(=LPS–)C57BL/6J and *in vitro* alternatively activated (IL-4) Mφ signatures, seven genes are up-regulated in common (Figure 1A, Supplementary Table 5): *Lpxn, Dhrs3, Mical1, Dnmt3a, Jun, Gab1*, and *P2ry1*. *Gab1, Jun*, and *P2ry1* are related to positive regulation of the MAPK cascade.

The Grb2-associated binder (Gab) proteins, which belong to the Gab/DOS family of docking proteins, function as essential elements in signal integration during the assembly of downstream signaling complexes (52). *Gab1* and *Gab2* (Gab1/2) are widely expressed in various cell types, including immune cells such as T cells, and mast cells. A recent study demonstrated that the deficiency of either Gab1 or Gab2 resulted in impaired M2 polarization (53). Gab1 regulates IL-4-induced macrophage polarization by activating AKT signaling (53). Another interesting gene is Jun Proto-Oncogene (*Jun*). The transcription factor family AP-1 is composed of homo- and heterodimeric complexes, which consist of Jun, Fos, activating transcription factor, and musculoaponeurotic fibrosarcoma proteins. These dimers are involved in different cellular processes, such as proliferation, apoptosis, and differentiation.

G Protein-Coupled Receptors (GPCRs) are important receptors able to regulate inflammation and immunity. P2Y1 (*P2ry1*) is an ADP receptor known to be expressed in several macrophages except for microglia, where it does not appear to be expressed (54). P2Y1 function in macrophages is not entirely understood. LPS decreases its expression *in vitro* (55).

The Glutamine synthetase (*Glul*) is a gene associated with glutamine metabolism, an essential pathway for the differentiation and function of macrophages (56). *Glul* indeed is known to be enhanced in M2 macrophages (56).

#### *In vivo* M2(=LPS–)C57BL/6J Signature Modulate Cell Metabolism Genes Activating Arginine and Lipid Catabolic Process

The *in vivo* M2(=LPS–)C57BL/6J signature includes 170 private genes not shared by *in vitro* classically or alternatively activated (IL-4) macrophages (Figures 1A,B, Supplementary Table 5).

As stated before, the M2 polarization exerts a switch on arginine metabolism and other metabolic pathways (57). The majority of genes present in the signature are indeed related to the modulation of the cell metabolism, i.e., lipid catabolic process. M2 polarized macrophages obtain their energy mostly from fatty acid oxidation and oxidative metabolism. The fatty acid oxidation process in *in vivo* M2(=LPS–)C57BL/6J macrophages is highlighted by the presence of several genes in the signatures such as Acyl-CoA Dehydrogenase (Long chain) and medium-chain (*Acadm*) (58). Carnitine Palmitoyltransferase 1A (*Cpt1a*) is also present in the signature, and it is associated with fatty acid beta-oxidation (59, 60).

The nuclear receptor PPARγ plays a dominant role in adipogenesis and storage of fatty acids as triglycerides in adipocytes. In macrophages, its activation enhances CD36 expression and increases the uptake of oxidized LDL and triacylglycerides (61). The nuclear receptor Pparγ activates several changes in the macrophage metabolism enhancing an M2-like response, and IL-4 is responsible for the first induction of its expression (62). The lipid modification and repair are another fundamental property in M2 macrophage function. This mechanism is mediated by the expression of *Acadm*, and other genes such as *B4galnt1, Hadh, Inpp5d, Soat1, Ip6k1, Hacl1, Echs1, Dgkz*, and *Hadh*b, present only in the *in vivo* M2 signature.

Other important cellular processes are post-translational modifications (PTM) that contribute to cell physiology by regulating protein stability, localization, and functions. Many of these modifications occur in response to LPS and regulate the interaction with environmental cues, being particularly relevant to macrophage functions. Several PTM genes were found in the M2(=LPS–)C57BL/6J signature. The ADP-ribosylating activity is one of them and is associated with the expression of poly(ADP-ribose)-polymerases (PARPs). *Parp1* it is present in M2(=LPS–)C57BL/6J macrophages and accounts for the majority of the poly(ADP-ribose) (PAR) polymer synthesis. Although enriched in the M2(=LPS–)C57BL/6J signature, *Parp1* has a pro-inflammatory function (63).

Another important PMT gene present in the M2(=LPS−) signature is the protein tyrosine phosphatase *Ptpn22*. Ptpn22 phosphatase activity is still not fully understood. Ptpn22 in macrophages skews the polarization toward M2 (64).

To define macrophage polarization, the field is in urgent need of good surface markers. At the mRNA level, we found *Cd300a*, a type I transmembrane protein with a long cytoplasmatic tail, known to regulate immune cells and macrophages (65, 66); and *Cd84*, a member of the SLAM family as candidates. CD84 is a cell surface receptor already known to be modulated during LPS stimulation (67). It is not known how CD300 or CD84 surface expression correlates with their mRNAs.

#### *In vitro* Alternatively Activated (IL-4) Mφ Solely Contain Standard IL-4 Induced M2 Markers

The *in vitro* alternatively activated (IL-4) Mφ signature contains 349 genes solely upregulated by IL-4 treatment *in vitro* (Figure 1A, Supplementary Table 5).

Among the genes up-regulated by IL-4 treatment *in vitro* we found the known M2 marker mannose receptor *Mrc1* also known as *Cd206*, as well as the tetraspanin *Cd9*, the TCR-associated molecule *Cd74, Bcl2, Arg1*, and the scavenger receptor *Cd36*.

It has been reported that several types of tissue-resident macrophages express *Cd206* in both mouse and humans (68). The depletion of mannose receptors has proven to increase the level of pro-inflammatory proteins (69). *Cd206* indeed promotes the expression of several anti-inflammatory cytokines and chemokines such as *Tgf* β, *Il10*, and *Ccl18* (the last also expressed in the signature), inducing a pro-fibrotic effect (68).

The tetraspanin CD9 is a cell surface glycoprotein, known to modulate cell adhesion and migration. In macrophages it has been found to regulate the LPS induced activation negatively; the loss of CD9 in CD9 knockout macrophages enhances LPS signaling (70). IFN-gamma signaling has been found to reduce CD9 expression in macrophages (71).

CD74 is also a cell-surface receptor for the cytokine macrophage migration inhibitory factor (MIF), mostly expressed and studied in B cells. MIF binding to CD74 induces its intramembrane cleavage and the release of its cytosolic intracellular domain, which regulates cell survival. CD74 with MIF also induces the expression and secretion of the cytokine, midkine. Midkine suppresses apoptosis by elevating the expression of Bcl-2 and inhibiting caspase 3 and 7 activity (72).

B Cell lymphoma 2 (*Bcl2*) is also expressed among the 350 up-regulated genes. *Bcl2* inhibits pro-apoptotic proteins (73). The lack of *Bcl2* expression in macrophages has been reported to accelerate the progression of atherosclerotic plaques in *Apoe*^−/−^ mice (74).

Other well-reported M2 macrophage markers are *Arg1* and *Cd36*. Arginase-1 is an enzyme of the urea cycle; its action catalyzes the hydrolysis of arginine to ornithine. Ornithine is the substrate for ornithine decarboxylase (ODC). This pathway regulates a multitude of cellular processes like DNA replication, protein translation, cell growth, and differentiation (75). Moreover, the absence of L-arginine was proven to reduce T-cell proliferation (76).

CD36 is a class B scavenger receptor for the endocytosis of triacylglycerol-rich lipoprotein particles, such as LDL and VLDL. It is also known to be an essential macrophage receptor for apoptotic cell recognition and phagocytosis (77).

Among the 36 down-regulated genes vs. untreated Mφ (Figure 1B) to be noted is the presence of genes commonly referred for cell cycle modulation such as *Ccnd1, Chek1, Cdc25b*, and *Ncapg2*.

### Mutual Gene Expression Among Opposite Signatures

#### *In vivo* M1(=LPS+)C57BL/6J Present Immunoregulatory Functions Shared With *in vitro* Alternatively Activated (IL-4) Mφ

In the signature comparison, 28 genes up-regulated in *in vivo* M1(=LPS+)C57BL/6J and *in vitro* alternatively activated (IL-4) Mφ signatures were found (Figure 1A, Supplementary Table 5), including four chemokines: *Ccl7, Ccl17, Ccl22*, and *Ccl24*.

*Ccl7* is known to be expressed by both M1 and M2 macrophages (78). CCL17 and CCL22 are both ligands of the CCR4 receptor. *In vitro* alternatively activated macrophages have been shown to produce all these chemokines in high amounts in the response of Th2 cytokines such as IL-4, and IL-13 (18, 79). Functionally, CCL17 and CCL22 together with CCL24 are potent chemoattractants, favoring the attraction of immune-inhibitory cells such as regulatory T-cells (Treg) (18). Another gene, type 1 myosin (*Myo1c*), related to cytoskeleton rearrangement, is fundamental for the migration and phagocytosis process of macrophages. Interestingly, TLR4 stimulation by LPS showed enrichment of cytoskeleton-associated proteins such as Myo1C among the LPS-regulated phosphopeptides (80). The cell surface markers CD44 (*Cd44*), and CD83 (*Cd83*) are also regulated in the different signatures. This is a good example illustrating that the cell surface phenotype observed *in vitro* alternatively activated (IL-4) Mφ cannot be perfectly translated into an M2 *in vivo* phenotype.

#### The *in vitro* Signature of Classically Activated (LPS+IFN-γ) Mφ Presents Apoptosis and Cell Death Genes Together With *in vivo* M2(=LPS–)C57BL/6J

Intriguingly, *in vivo* M2(=LPS–)C57BL/6J and *in vitro* classically activated (LPS+IFN-γ) Mφ shared the up-regulation of seven genes; *Fos, Tuba4a, Tnfrsf21, Sdc1, Ggta1, Rhov*, and *Psen2* (Figure 1A, Supplementary Table 5).

Presenilin 2 (*Psen2*) is the catalytic core component of protease complexes that mediate regulated intramembrane proteolysis of their substrates. The lack of normal Psen2 function has been associated with diminished LPS-induced macrophage responsiveness (81). Another modulated gene is Syndecan1 (*Sdc1*), that is related to the wound healing process (82, 83). A TNF receptor family gene (*Tnfrsf21*) is among these seven in the M2(=LPS–)C57BL/6J and classically activated Mφ *in vitro* (Supplementary Table 5). Activation of TNFRSF receptors induces the apoptotic process through the initiation of specific receptors (so-called Death Receptors). The best-known example is Fas (*Tnfrsf6*) (84). These genes, indeed, can activate the death effector domain leading to caspases activation and cell death (85).

## Pathway Analysis Overview

The gene lists for the different macrophage signatures were further analyzed by Ingenuity Pathways Analysis (IPA). The enriched canonical pathways for each signature with a *p*-value cutoff of 0.001 are reported in Supplementary Table 6.

**Figure 2:**
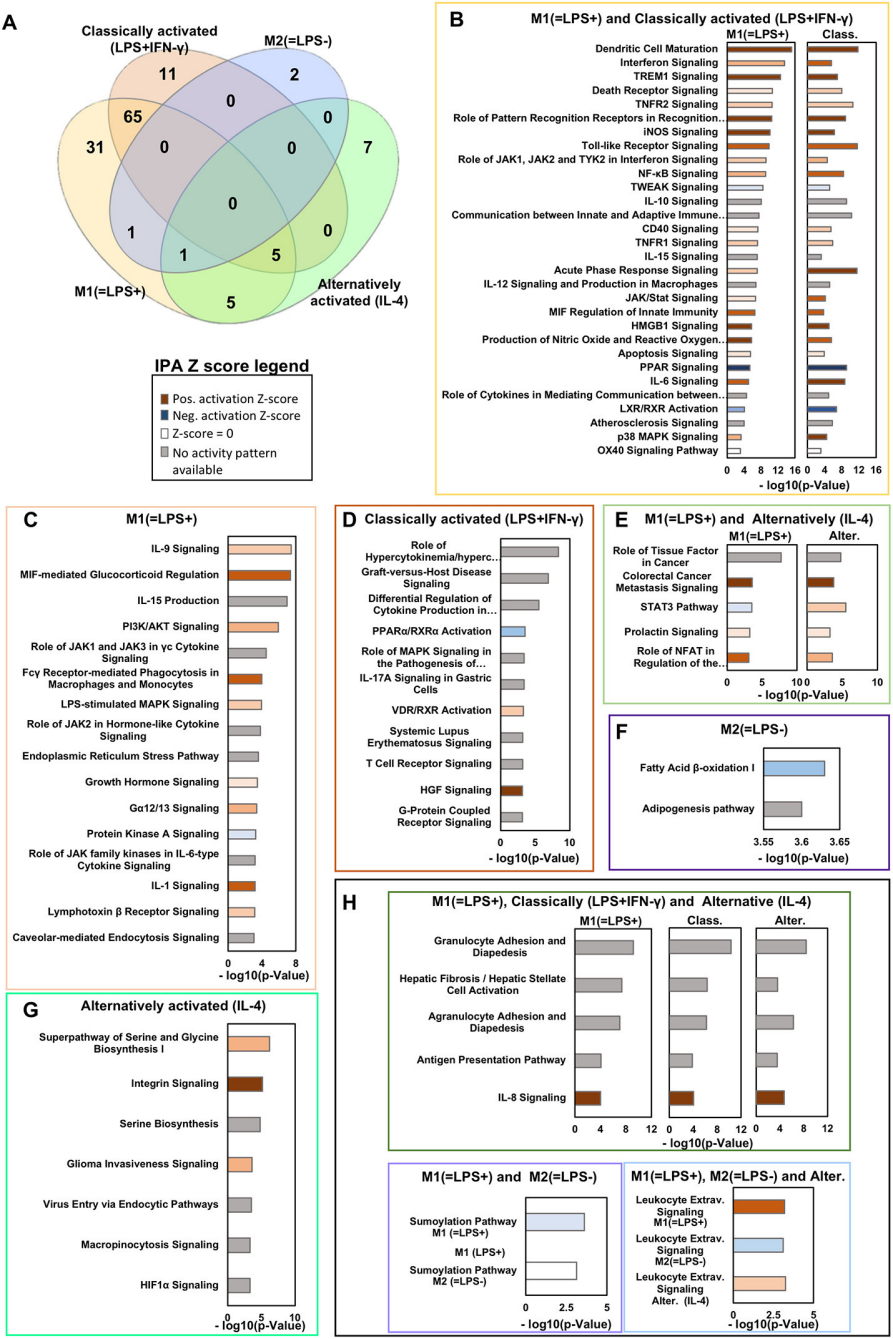
Ingenuity pathways analysis *in vivo* M1(=LPS+)C57BL/6J, M2(=LPS–)C57BL/6J, and *in vitro* classically (LPS+IFN-γ) or alternatively activated (IL-4) macrophages. **(A)** Venn diagram showing the key canonical pathways enriched *in vivo* M1(=LPS+)C57BL/6J, M2(=LPS–)C57BL/6J, M2(=LPS–)C57BL/6J, classically activated (LPS+IFN-γ) and alternatively activated (IL-4) macrophage signatures as determined by Ingenuity pathway analysis (IPA). The number of pathways is sorted by a *P*-value cutoff of 0.001. **(B–H)** Selected canonical pathways ranked based on –log(*P*-value) divided as reported in the Venn diagram are shown in the boxes. The Z-score of each pathway is reported by the color of the bars (see legend). Light/Dark shades represent smaller/larger absolute values of Z-score.

The enriched pathways for *in vivo* macrophage signatures M1(=LPS+)C57BL/6J, M2(=LPS–)C57BL/6J and the *in vitro* classically activated (LPS+IFN-γ) and alternatively activated (IL-4) signatures were then analyzed and compared using the Venny 2.1 online tool (Figure 2A, Supplementary Table 7). The z-score is a prediction on the state of the pathway based on the expression level of the genes associated with that pathway. A positive z-score implies that the pathway is activated and a negative Z-score implies that the pathway is inhibited. z-score < -2 or z-score >2 was considered significant. The genes enriched for each pathway and the corresponding z-score are fully reported in Supplementary Table 8. Reassuringly, the largest number, 65 pathways are shared between the *in vivo* M1(=LPS+)C57BL/6J and *in vitro* classically activated (LPS+IFN-γ) macrophages (Figure 2A). Our analysis shows 31 pathways that are private to *in vivo* M1(=LPS+)C57BL/6J and two pathways private to *in vivo* M2(=LPS–)C57BL/6J (Figure 2A). *In vitro* alternatively and classically activated Mφ show 7 and 11 private pathways, respectively. Interestingly, no pathways are commonly modulated by *in vivo* M2(=LPS–)C57BL/6J and *in vitro* alternatively activated (IL-4) Mφ, suggesting that these macrophage phenotypes are very different from each other. Instead, five pathways are shared by the *in vivo* M1(=LPS+)C57BL/6J and *in vitro* alternatively activated (IL-4) Mφ. Another five are shared between M1(=LPS+)C57BL/6J, classically and alternatively activated (IL-4) Mφ (Figure 2A).

### *In vivo* M1(=LPS+)C57BL/6J and *in vitro* Classically Activated (LPS+IFN-γ) Mφ Common Pathways

*In vivo* M1(=LPS+)C57BL/6J and *in vitro* classically activated (LPS+IFN-γ) Mφ present 65 pathways in common (Supplementary Table 7). The top 30 are shown in Figure 2B. The top modulated pathways by *p*-value include Dendritic Cell Maturation, showing a close relationship between M1 macrophages, and mature dendritic cells.

The Dendritic Cell Maturation pathway is a big pathway that includes ≈190 genes. Among these genes many are commonly activated during M1 like macrophage polarization such as *Csf2, Il12, Il18, Il15, Il23* etc. (the full list of genes found inside this pathway for M1(=LPS+) and classically activated Mφ is reported in Supplementary Table 8). A prominent inflammatory DC is the TIP-DC, producing TNF and iNOS (86).

Next is Interferon Signaling, driven by the genes *Irf1, Irf9, Jak2, Stat1, Ifnar2, Stat2, Ifi35, Ptpn2, Ifitm3, Tap1, Psmb8*, and *Rela*. Trem1 Signaling is also highly significant. Death Receptor Signaling, OX40 Signaling Pathway and TNFR2 Signaling are related and contain many of the TNF receptor superfamily signaling pathways. The pathway Role of Pattern Recognition Receptors in Recognition of Bacteria and Viruses contains TLRs (also in the pathway Toll-like Receptor Signaling) and other receptors. iNOS Signaling is prominently enriched; iNOS is the main bacterial killing pathway employed by M1 and classically activated macrophages.

The z-scores given for iNOS signaling in M1(=LPS+) and classically activated (LPS+IFN-γ) Mφ present comparable values with a slightly lower p-value in M1(=LPS+) than classically activated macrophages. Muller et al. previously reported that classically activated Mφ (LPS+IFN-γ) produce more *Nos2*, than LPS activated macrophages (87, 88). The z-score is a prediction on the state of the pathway based on the expression level of the several genes associated with that pathway but is not reliable for single gene expression. In this particular case the genes modulated inside the iNOS signaling pathway by M1(=LPS+) signature were slightly different compared to the *in vitro* classically activated (LPS+IFN-γ) macrophage signature as reported in Supplementary Table 8.

Related to this is the Production of Nitric Oxide and Reactive Oxygen Species in Macrophages. Next is the Role of JAK1, JAK2, and TYK2 in Interferon Signaling and NF-κB Signaling. Unexpectedly, TWEAK Signaling ranks highly (*p* = 3 × 10^−9^). TWEAK is an often-overlooked member of the TNF receptor superfamily. Two other TNF receptor superfamily pathways are CD40 Signaling and TNFR1 Signaling. The anti-inflammatory IL-10 Signaling pathway is also prominent, showing that an anti-inflammatory program is detectable in the generally pro-inflammatory *in vivo* M1(=LPS+)C57BL/6J and *in vitro* classically activated (LPS+IFN-γ) macrophages. Communication between Innate and Adaptive Immune Cells shows that activated Mφ also instruct adaptive immunity. Related to this is the Role of Cytokines in Mediating Communication between Immune Cells pathway. IL-15 Signaling is a pathway related to IL-2. IL-15 signaling stimulates T cell proliferation and inhibits IL-2-mediated activation-induced cell death (89). Acute Phase Response Signaling is normally expected in liver cells, but also active in *in vivo* M1(=LPS+)C57BL/6J and *in vitro* classically activated classically activated (LPS+IFN-γ) Mφ. IL-12 Signaling and Production is a defining proinflammatory property of macrophages. JAK/Stat Signaling covers many cytokine signaling pathways. MIF Regulation of Innate Immunity focuses on an atypical chemokine, MIF, with powerful effects in innate immunity. HMGB1 Signaling is also pro-inflammatory and together with CD14 mediates the activation of TLR4 (90).

Significant enrichment of Apoptosis Signaling shows the precarious situation of these highly activated macrophages that teeter on the edge of oblivion, as is necessary for terminating the aggressive phase of inflammation. Related to this is activation of the LXR/RXR Activation pathway, which tends to be anti-inflammatory. PPAR Signaling is related to metabolic effects. IL-6 Signaling is its own module, which is of key importance in cardiovascular inflammation, suggesting that *in vitro* classically activated (LPS+IFN-γ) and *in vivo* M1(=LPS+)C57BL/6J macrophages share the ability to promote atherosclerosis. Indeed, the Atherosclerosis Signaling pathway is also significantly enriched.

### *In vivo* M1(=LPS+)C57BL/6J Private Pathways

Some pathways are private to *in vivo* M1(=LPS+)C57BL/6J (Figure 2C, Supplementary Table 7). They include IL-9 Signaling, IL-15 Production (although a related pathway is found in both *in vivo* M1(=LPS+)C57BL/6J and classically activated Mφ), and PI3K/AKT Signaling. The Role of JAK1 and JAK3 in common gamma chain (γc) Cytokine Signaling shows than common gamma chain cytokine pathways are more enriched *in vivo* M1(=LPS+)C57BL/6J macrophages. Interestingly, Fcγ Receptor-mediated Phagocytosis in Macrophages and Monocytes is only enriched in *in vivo* M1(=LPS+)C57BL/6J and not *in vitro* classically activated (LPS+IFN-γ) Mφ, as is LPS-stimulated MAPK Signaling. *In vivo* M1(=LPS+)C57BL/6J also have a signature for Endoplasmic Reticulum Stress Pathway. Enriched Gα12/13 Signaling suggests activation of chemokine receptor signaling. Protein Kinase A Signaling is an anti-inflammatory pathway. This pathway has a negative z-score, meaning it is likely suppressed *in vivo* M1(=LPS+)C57BL/6J macrophages. The pathway Role of JAK family kinases in IL-6-type Cytokine Signaling is similar to the IL-6 pathway mentioned above, which is shared between *in vitro* classically activated (LPS+IFN-γ) Mφ and M1(=LPS+)C57BL/6J. Of great interest for cardiovascular disease, IL-1 Signaling is private to the *in vivo* M1(=LPS+)C57BL/6J signature and not enriched in *in vitro* classically activated (LPS+IFN-γ) Mφ. Finally, the Lymphotoxin β Receptor Signaling is present in M1(=LPS+)C57BL/6J; it is a TNF receptor superfamily member involved in organizing lymphoid tissues believed to control pro-inflammatory response reactions (91). Also this pathway suggests that *in vivo* M1(=LPS+)C57BL/6J macrophages keep control of inflammation avoiding harmful hyper reactivity with a balance of pro and anti-inflammatory stimuli.

### *In vitro* Classically Activated (LPS+IFN-γ) Mφ Private Pathways

*In vitro* classically activated (LPS+IFN-γ) Mφ show 11 private pathways (Figure 2D). The top modulated pathways by p-value include Role of Hypercytokinemia/Hyperchemokinemia and Graft Vs. Host Disease Signaling, both driven by a similar set of cytokines including *Il18, Il1rn, Il1b, Tnf, Il1a*, and *Il6* (Supplementary Table 3). The third highly significant modulated pathway is Differential Regulation of Cytokine Production in Macrophages and T Helper Cells by IL-17A and IL-17F, driven by the modulation of *Il12a, Il1b, Ccl5, Tnf, Il12b*, and *Il6*.

The involvement of IL-17 signaling in *in vitro* classical Mφ activation is also suggested by the IL-17A Signaling pathway driven by, among other genes, *Il17ra*. The HGF Signaling pathway is also induced. HGF is a well-known motogenic factor, inducing directional migration, and differentiation of monocytes (92). By contrast, the PPARα/RXRα Activation pathway is negatively modulated (Figure 2D). The negative regulation of this pathway might be linked with switching off lipid metabolism and boosting the glycolytic pathway (93).

### *In vivo* M2(=LPS–)C57BL/6J and Alternatively Activated (IL-4) Mφ Pathways

Intriguingly, no pathways were regulated in common among the M2(=LPS–)C57BL/6J *in vivo* and *in vitro* alternatively activated (IL-4) Mφ signature at the *p*-value cut-off applied (Figure 2E).

### *In vivo* M2(=LPS–)C57BL/6J Pathways

The two pathways privately expressed *in vivo* M2(=LPS–)C57BL/6J are the adipogenesis pathway and fatty acid synthesis pathway, both involved in anabolic processes (Figure 2F). Opposed to the *in vivo* M1(=LPS+)C57BL/6J activation, *in vivo* M2(=LPS–)C57BL/6J regulated gene transcription occurs in conditions favoring mitochondrial metabolism and oxidative glucose metabolism, with an increase of anabolic process that properly corresponds with the *in vivo* M2 functions (94, 95).

### *In vitro* Alternatively Activated (IL-4) Mφ Pathways

The *in vitro* alternatively activated (IL-4) Mφ show seven private pathways (Figure 2G). Serine Biosynthesis and Serine and Glycine Biosynthesis I are two of the top three enriched pathways by p-value, confirming that *in vitro* alternatively activated (IL-4) Mφ polarization involves coordinated metabolic and transcriptional rewiring. The third top modulated pathway by p-value is the Integrin Signaling pathway (Figure 2G), suggesting the activation of the molecular machineries responsible of tissue infiltration. Integrins allow the infiltration of macrophages and other cells that start the removal of necrotic tissue and initiate the tissue regeneration processes (96, 97).

### Pathways Shared Among Opposite Signatures

Some pathways are common among opposite signatures. Specifically, we found 11 pathways shared between *in vivo* M1(=LPS+)C57BL/6J and *in vitro* alternatively activated (IL-4) Mφ, with five of them also present *in vitro* classically activated (LPS+IFN-γ) Mφ (Figure 2H). The top two regulated pathways common for all three signatures are the Granulocyte Adhesion and Diapedesis and the Agranulocyte Adhesion and Diapedesis pathways that are driven by several chemokines and adhesion molecules including *Ccl2, Cxcl9, Ccl7, Ccl24, Ccl22, Ccl9, Cxcl16, Ccl17, Icam1*, and *Vcam1* (Supplementary Table 3).

The IL-8 signaling pathway has a high Z score *in vivo* M1(=LPS+)C57BL/6J, and *in vitro* classically and alternatively activated (IL-4) Mφ. This pathway is known to support acute inflammation inducing the active neutrophil recruitment (98).

Exclusive for *in vivo* M1(=LPS+)C57BL/6J and *in vitro* alternatively activated (IL-4) Mφ, we found the Role of Tissue Factor pathway, driven by the expression of *Hbegf, Hck, Csf2, Src, Itgav, Jak2, Plaur, F10, Egr1, Mmp13, Rps6ka2, Stat5a, Csf1*, and *Rps6ka4* (Supplementary Table 3).

The STAT3 pathway showed an opposite Z score between *in vitro* alternative Mφ and *in vivo* M1(=LPS+)C57BL/6J (Figure 2H). The other three pathways instead show similar positively regulation among the two signatures (Figure 2H).

*In vivo* M1(=LPS+)C57BL/6J, M2(=LPS–)C57BL/6J and *in vitro* alternatively activated (IL-4) Mφ shared the Leukocyte extravasation signaling pathway.

Among the *in vivo* M1(=LPS+)C57BL/6J, and M2(=LPS–)C57BL/6J only the sumoylation pathway is shared, but presumably downregulated by the M1(=LPS+)C57BL/6J (Figure 2H).

## *In vivo* M(LPS±) Signatures Better Predict Survival in Cancer Biopsy Transcriptome of Pre-chemotherapy Biopsies From Osteosarcoma Patients

To address the question whether *in vivo* M(LPS±) or *in vitro* classically/alternatively activated (IL-4) gene signatures can predict cancer survival, we used the PRECOG database that ranks genes by overall tumor survival.

**Figure 3:**
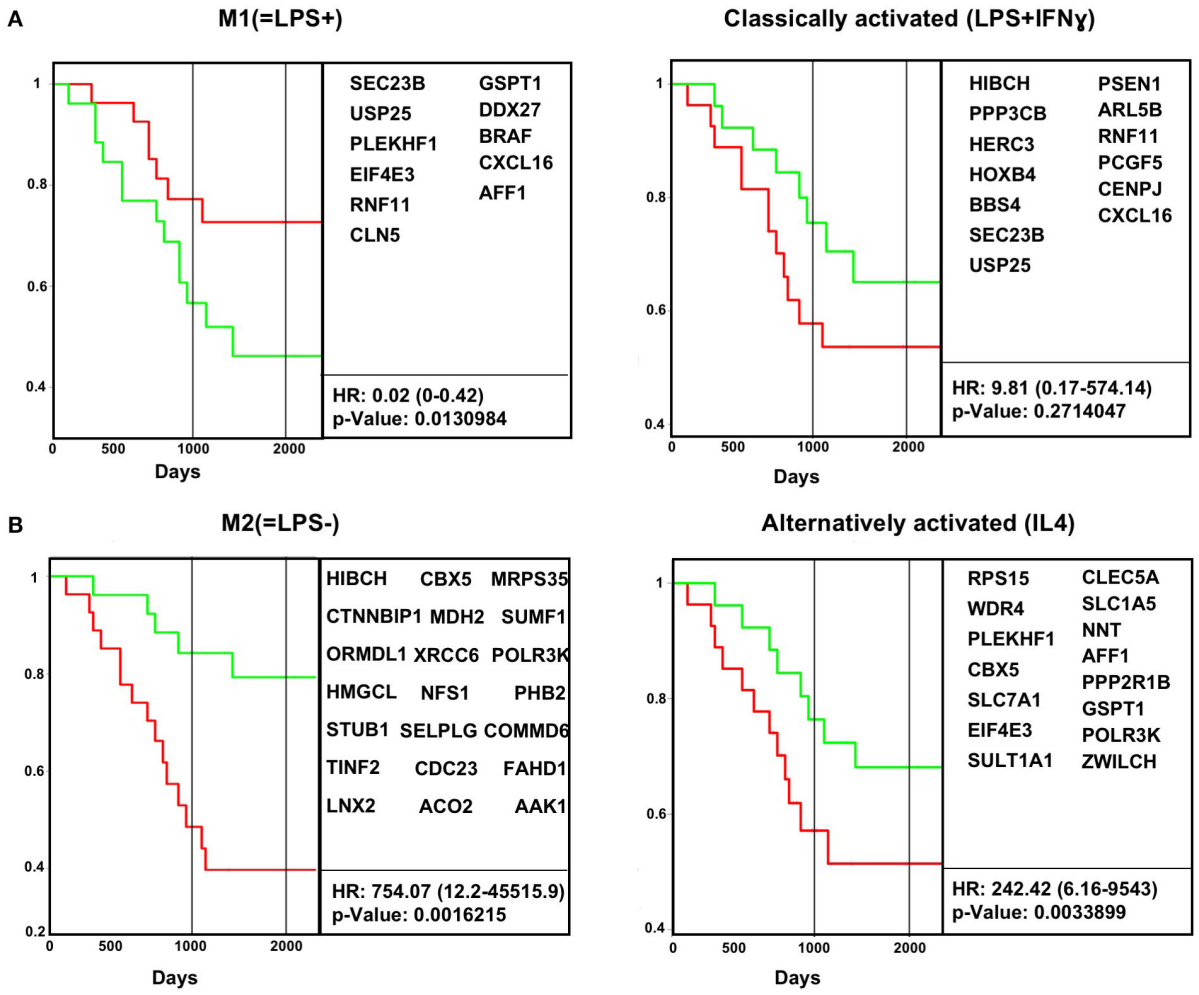
*In vivo* macrophage signatures predict survival in osteosarcoma cancer biopsy transcriptomes. Survival data for human osteosarcoma cancer biopsies (GSE21257) were analyzed for the impact of M1(=LPS+) and classically activated (LPS+IFN-γ) **(A)** and M2(=LPS−) and alternatively activated (IL-4) **(B)** gene expression signatures in the tumor biopsy transcriptome. Kaplan–Meier curves were plotted using ProggeneV2, divided by the median of the mean expression of a tumor-specific gene list (in boxes). Hazard ratio (HR, cox proportional hazard analysis) and significance (log rank *P*-value) are shown. Red, green curves indicate high, low expression of the respective signature genes. The two vertical black lines indicates 3 and 5 years, respectively.

As proof of concept, survival data from Buddingh et al. human osteosarcoma (GSE21257) was analyzed for the impact of the original *in vivo* M1(=LPS+) and *in vitro* classically activated (LPS+IFN-γ) and *in vivo* M2(=LPS–) and *in vitro* alternatively activated (IL-4) gene expression signatures in the tumor biopsy transcriptome from pre-chemotherapy biopsies of osteosarcoma patients. The genes applied in the analysis were extracted based on Cox linear regression analysis from the original signatures. Only the genes with a *p*-value < 0.01 from the *in vitro* and *in vivo* macrophages signatures were subsequently used to analyze survival in the published osteosarcoma datasets retrospectively (GSE21257) (Figure 3, Supplementary Table 9). Kaplan–Meier curves were plotted using ProggeneV2, divided by the median of the mean expression of a tumor-specific gene list.

We find the *in vivo* M1(=LPS+) signature correlates positively with survival, which means patients with tumors with above median expression of the M1(=LPS+) signature genes survived significantly longer (Figure 3A). Conversely, enrichment of M2(=LPS–) signature genes correlates with cancer death (Figure 3B). The *in vitro* classical M1 signatures did not correlate with survival. The *in vitro* alternative M2 gene signature correlates with cancer death, but less than the M2(=LPS–) *in vivo* signature.

*In vitro* evidence on BMDMs suggest that activation with two molecular signals from the microenvironment is required for efficient induction of M1 like phenotype in murine macrophages as defined by tumoricidal activity, NO production, and secretion of pro-inflammatory and Th1-polarizing cytokines (87).

However, it is not clear whether cytokines or other cancer therapies can shift macrophages to M1 in an individual with genetic predisposition for M2. M1 polarizers show preclinical evidence of improving cancer outcomes (99, 100). M1/M2 is also important in other diseases like atherosclerosis (101) and autoimmune diseases.

## Concluding Remarks

Several efforts have been made trying to define the molecular networks underlying polarized activation of macrophages. This review highlights the difference between cytokine effects on BMDMs *in vitro* vs. the response of peritoneal macrophages to LPS. Transcriptomes of LPS positive responder [M1(=LPS+)] vs. negative responder [M2(=LPS–)] macrophages show some overlap with *in vitro* classically (LPS+IFN-γ) vs. alternatively activated (IL-4) BMDMs. However, the majority of regulated genes are different. Thus, *in vivo* M1(=LPS+) and M2(=LPS–) macrophages and *in vitro* classically (by LPS, IFN-γ) and alternatively activated (by IL-4) Mφ are not comparable. Some genes and pathways are even shared between the *in vivo* and the *in vitro* opposite signature.

Bone marrow contains immature macrophage precursors, which are not found in peripheral tissues like the peritoneum, so BMDMs are a mix of monocyte-derived and precursor-derived macrophages. We show that the *in vivo* M1(=LPS+) and M2(=LPS–) gene signatures are also applicable to human transcriptomes. Human alveolar macrophages for example expressed about 70% of the mouse-derived gene signatures. Human monocyte-derived macrophages (hMDM) transcriptome (102) in response to LPS stimuli expressed about 57% of the M1(=LPS+) gene signature and only 37% of the *in vitro* classically activated (LPS+IFN-γ) signature (Supplementary Table 10). We also demonstrated an increased enrichment score for the *in vivo* M1(=LPS+) signature in isolated human synovial macrophages from rheumatoid arthritis patients (22). The *in vivo* M2(=LPS–) signature instead presented a high enriched score in isolated human synovial macrophages from healthy donors (22).

In conclusion, classical and alternative macrophage activation *in vitro* does not match the *in vivo* M1/M2 polarization. This fundamental discrepancy explains why most surface markers identified on *in vitro* generated macrophages fail to translate to macrophages *in vivo*. We propose that a new effort is needed to discover valid M1/M2 markers, which will help understand macrophage behavior *in vivo*.

## Author Contributions

KL and MO wrote the paper. MO, YG, and AP performed the analysis, constructed the Venn diagrams, and input to the Supplementary Tables.

### Conflict of Interest Statement

The authors declare that the research was conducted in the absence of any commercial or financial relationships that could be construed as a potential conflict of interest.
