# Corrigendum: Macrophage Polarization: Different Gene Signatures in M1(LPS+) vs. Classically and M2(LPS–) vs. Alternatively Activated Macrophages

**DOI:** 10.3389/fimmu.2020.00234

**Published:** 2020-02-25

**Authors:** Marco Orecchioni, Yanal Ghosheh, Akula Bala Pramod, Klaus Ley

**Affiliations:** ^1^Division of Inflammation Biology, La Jolla Institute for Immunology, La Jolla, CA, United States; ^2^Department of Bioengineering, University of California, San Diego, La Jolla, CA, United States

**Keywords:** macrophage, innate immunity, M1, M2, cancer

In the original article, there was a mistake in Figure 3 as published. Three gene names were misspelled in the previous version. In Figure 3A, M1(=LPS+) panel, the correct gene name for EIFE3 is EIF4E3. In Figure 3B, alternatively activated (IL-4) panel, the correct gene name for WRD4 is WDR4. Also in Figure 3B, the correct gene name for POLRR3K is POLR3K. The corrected Figure 3 appears below.

Additionally, in the original article, there was a mistake in the legend for Figure 3 as published. “M1(=LPS+)” was written as “M1(LPS+)”, and “M2(=LPS−)” was written as “M2(LPS−)”. The correct legend for Figure 3 appears below.

**Figure 3:**
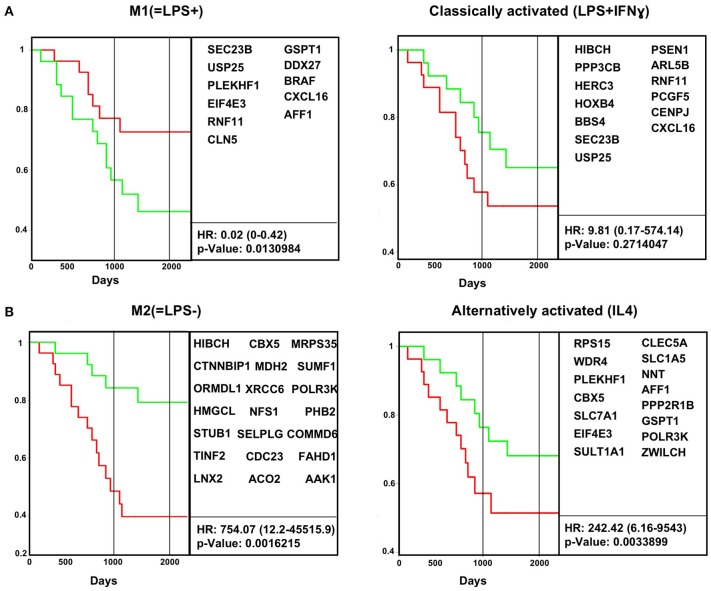
*In vivo* macrophage signatures predict survival in osteosarcoma cancer biopsy transcriptomes. Survival data for human osteosarcoma cancer biopsies (GSE21257) were analyzed for the impact of M1(=LPS+) and classically activated (LPS+IFN-γ) **(A)** and M2(=LPS−) and alternatively activated (IL-4) **(B)** gene expression signatures in the tumor biopsy transcriptome. Kaplan–Meier curves were plotted using ProggeneV2, divided by the median of the mean expression of a tumor-specific gene list (in boxes). Hazard ratio (HR, cox proportional hazard analysis) and significance (log rank *P*-value) are shown. Red, green curves indicate high, low expression of the respective signature genes. The two vertical black lines indicates 3 and 5 years, respectively.

The authors apologize for these errors and state that this does not change the scientific conclusions of the article in any way. The original article has been updated.

